# Serotonin is an endogenous regulator of intestinal CYP1A1 via AhR

**DOI:** 10.1038/s41598-018-24213-5

**Published:** 2018-04-17

**Authors:** Christopher Manzella, Megha Singhal, Waddah A. Alrefai, Seema Saksena, Pradeep K. Dudeja, Ravinder K. Gill

**Affiliations:** 1 Division of Gastroenterology & Hepatology, University of Illinois at Chicago, Chicago, IL United States; 2 Jesse Brown VA Medical Center, Chicago, IL United States; 3 Department of Physiology & Biophysics, University of Illinois at Chicago, Chicago, IL United States

## Abstract

Aryl hydrocarbon receptor (AhR) is a nuclear receptor that controls xenobiotic detoxification via induction of cytochrome P450 1A1 (CYP1A1) and regulates immune responses in the intestine. Metabolites of L-tryptophan activate AhR, which confers protection against intestinal inflammation. We tested the hypothesis that serotonin (5-HT) is an endogenous activator of AhR in intestinal epithelial cells. Treatment of Caco-2 monolayers with 5-HT induced *CYP1A1* mRNA in a time- and concentration-dependent manner and also stimulated CYP1A1 activity. CYP1A1 induction by 5-HT was dependent upon uptake via serotonin transporter (SERT). Antagonism of AhR and knockdown of AhR and its binding partner aryl hydrocarbon receptor nuclear translocator (ARNT) attenuated CYP1A1 induction by 5-HT. Activation of AhR was evident by its nuclear translocation after 5-HT treatment and by induction of an AhR-responsive luciferase reporter. *In vivo* studies showed a dramatic decrease in CYP1A1 expression and other AhR target genes in SERT KO ileal mucosa by microarray analysis. These results suggest that intracellular accumulation of 5-HT via SERT induces CYP1A1 expression via AhR in intestinal epithelial cells, and SERT deficiency *in vivo* impairs activation of AhR. Our studies provide a novel link between the serotonergic and AhR pathways which has implications in xenobiotic metabolism and intestinal inflammation.

## Introduction

The aryl hydrocarbon receptor (AhR) is an evolutionarily conserved nuclear receptor that is widely expressed in multiple organs including brain, liver, lung, and the gastrointestinal (GI) tract^1,2^. Once activated, AhR translocates to the nucleus and dimerizes with co-factors including aryl hydrocarbon receptor nuclear translocator (ARNT), and binds to xenobiotic-responsive units (XREs) to increase expression of AhR-responsive genes. The canonical gene targets of AhR are the cytochrome P450, family 1 enzymes including CYP1A1, which are involved in the metabolism of polycyclic aromatic hydrocarbons and other xenobiotics^3,4^. Over recent years, new gene targets of AhR have been established that are involved in diverse physiological processes including development, hematopoiesis, and immune modulation^1,2,5^. In the gut, AhR is important for maintaining immune cell populations, forming tertiary lymphoid follicles, and inducing proliferation of colonic stem cells^6,7^.

Increasing evidence has established new roles for AhR beyond acting as a xenobiotic sensor, particularly as a regulator of inflammatory pathways^2,7,8^. Several reports have demonstrated that administration of AhR agonists markedly attenuated experimentally induced colitis in mice^9,10^. Conversely, AhR KO mice exhibited a disrupted intestinal homeostasis and are more susceptible to experimental colitis^6,7,9,11^. Mice with epithelial AhR deficiency, but not those with macrophage-specific or T-cell specific AhR deficiency, were more sensitive to DSS-induced colitis with increased apoptosis in intestinal epithelia^8^.

Xenobiotic ligands including dioxin and polycyclic aromatic hydrocarbons were among the first ligands to be discovered for AhR^12^. Endogenous ligands of AhR have been proposed in the form of tryptophan metabolites such as kynurenine, cinnabarinic acid, and 6-formylindolo[3,2*b*]carbazole (FICZ)^12^. However, the proposed endogenous ligands have not yet been shown to exist *in vivo* at concentrations sufficient enough to activate AhR under normal conditions (12). Bacteria-derived tryptophan metabolites such as indole, indoleacetic acid, 3-methylindole, and tryptamine are established AhR ligands, suggesting that AhR is a mediator of communication between tryptophan-metabolizing bacteria and the host^13–15^. While tryptophan metabolites have been shown to activate AhR, the involvement of serotonin (5-hydroxytryptamine, 5-HT) and serotonergic machinery in AhR signaling has not been investigated.

5-HT is a tryptophan-derived neurotransmitter and hormone that plays an important role in regulating diverse physiological processes in both the brain and the gut. In the GI tract, 5-HT modulates electrolyte secretion and absorption, blood flow, perception of nausea or pain, and intestinal motility^16–18^. The GI tract is a major source of 5-HT, with 95% of the whole body 5-HT being synthesized by specialized intestinal epithelial cells called enterochromaffin (EC) cells^19^. EC cells release 5-HT into the intestinal lumen and the lamina propria where it can bind to several subtypes of 5-HT receptors (5-HTRs) to elicit its various physiological actions^18^. Extracellular 5-HT is internalized by the serotonin transporter (SLC6A4, SERT), which transports 5-HT into the cell with high affinity via a Na^+^ and Cl^−^ dependent process^20^. Once inside the cell, monoamine oxidases degrade 5-HT into 5-hydroxyindoleacetic acid (5-HIAA), which is conjugated for excretion^20^. Indeed, mice lacking SERT exhibit pleotropic phenotypes including increased anxiety-like behavior, abnormal GI motility, obesity, and insulin resistance^21,22^. Decreased SERT expression and consequent high extracellular 5-HT levels have been implicated in several pathophysiological conditions such as inflammatory bowel disease (IBD) and irritable bowel syndrome (IBS)^23–25^. In addition, SERT deficiency increases the susceptibility of mice to colitis in IL-10 deficient mice^26^ as well as in 2,4,6-trinobenzenesulfonic acid (TNBS) treated mice^27^. However, how SERT deficiency exacerbates severity of intestinal inflammation is not known.

Our current findings demonstrate for the first time that 5-HT can induce CYP1A1 expression via AhR in intestinal epithelial cells, and that this activation is dependent upon its uptake into the cell via SERT. Expression of *Cyp1a1* in the intestinal mucosa of SERT KO mice was dramatically reduced, which paralleled our *in vitro* findings. Microarray analysis of SERT KO ileal mucosa revealed differential expression of other AhR targets including chemokine *Ccl20* and the growth factor *Areg*^28,29^. The well-established IBD susceptibility gene *Tnfsf15* was also differentially regulated in the SERT KO mice^30–32^. As 5-HT does exist in sufficient concentrations in the GI tract to carry out its many functions, these data have novel physiological implications and may explain the increased susceptibility of SERT KO to inflammation via impairment of AhR pathways. Furthermore, as AhR is a receptor for environmental components such as combustion products in cigarette smoke, dioxin-like toxins, and bacterial products; our studies reveal a novel link by which serotonergic signaling can influence the contribution of the environmental agents to gastrointestinal diseases such as IBD.

## Results

### 5-HT induces *CYP1A1* mRNA expression and CYP1A1 activity in intestinal epithelial cells

**Figure 1:**
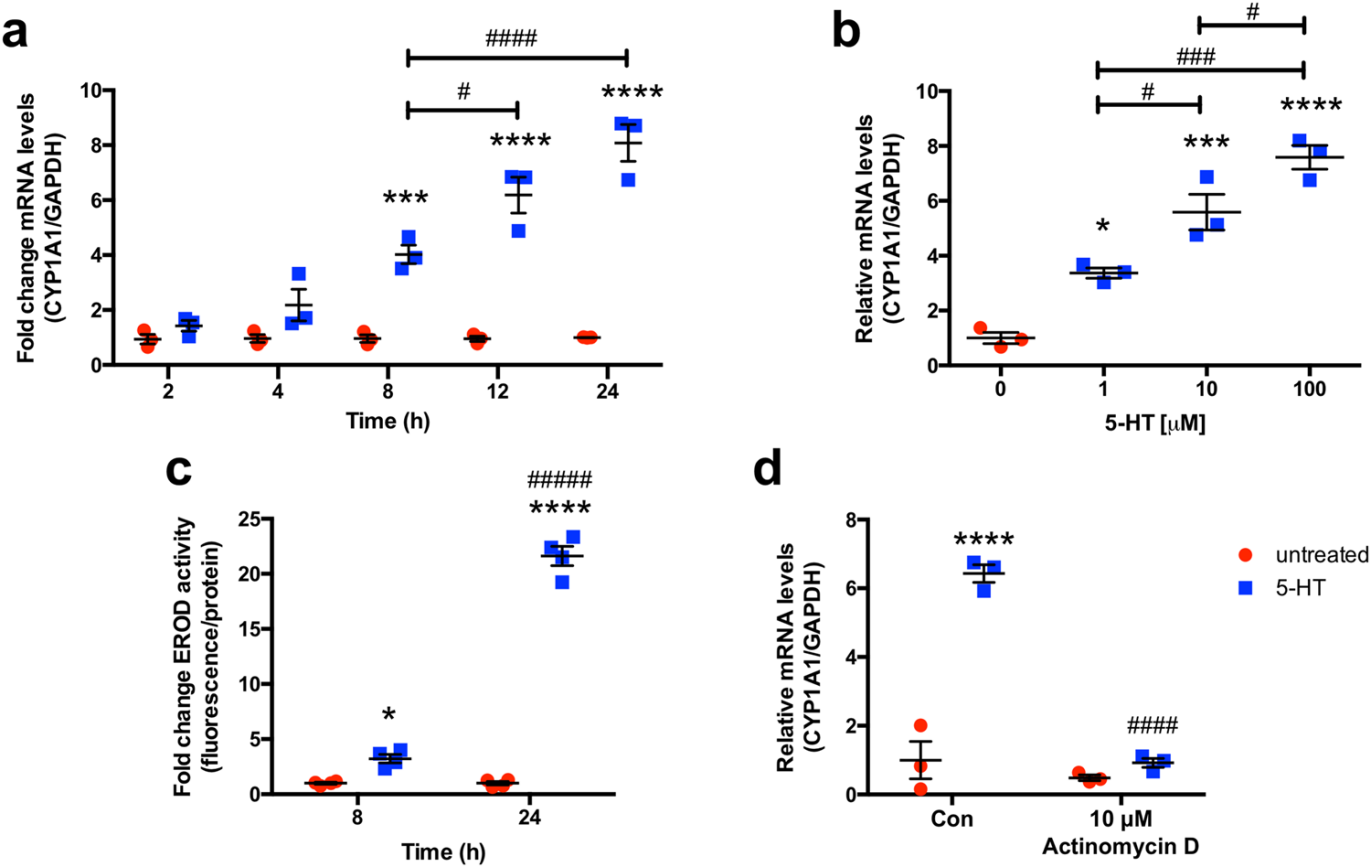
5-HT induces CYP1A1 mRNA expression and activity via a transcriptional mechanism. Caco-2 cells were plated at low density and allowed to differentiate for 10–14 d in medium containing 20% serum before treatments were performed. (**a**) Caco-2 cells were treated with 5-HT in serum-free culture medium at a concentration of 10 μM for 2 h–24 h (*n* = 3) (**b**) or for 8 h at a concentration range of 1 μM–100 μM (*n* = 3). Data represent the relative expression of *CYP1A1* mRNA quantified by qPCR as compared to time-matched untreated cells. (**c**) Caco-2 cells were treated with 10 μM 5-HT in serum-free culture medium and CYP1A1 activity was measured by the ethoxyresorufin-O-deethylase (EROD) assay (*n* = 4). Assays were performed using triplicate wells for each treatment and values were normalized to total protein by Bradford assay. Results are expressed as fold-change over the activity of time-matched untreated cells. (**d**) Caco-2 cells were treated with vehicle (Con) or actinomycin D for 1 h before co-treatment with 5-HT for 24 h in serum-free culture medium (*n* = 3). Data analyzed by 1-way (**a**) or 2-way (**b**–**d**) ANOVA followed by Tukey’s multiple comparisons test. *P < 0.05, ***P < 0.001, ****P < 0.0001 vs. no 5-HT. ^#^*P* < 0.05, ^###^*P* < 0.001, ^####^*P* < 0.001 between groups. Red circle = untreated, blue square = 5-HT.

Our initial studies examined the effects of 5-HT on *CYP1A1* mRNA levels utilizing model intestinal epithelial cell line Caco-2. Time-course of 5-HT (10 μM) demonstrated a significant induction in *CYP1A1* mRNA expression as early as 8 h with 8-fold induction after 24 h (Fig. 1a). The concentration-dependence of 5-HT treatment of Caco-2 cells at 8 h was next investigated. 5-HT was able to induce *CYP1A1* mRNA expression at concentrations as low as 1 μM, and the effect was concentration-dependent (Fig. 1b). After 8 h, 10 μM 5-HT induced *CYP1A1* mRNA nearly 6-fold, while 100 μM 5-HT induced by *CYP1A1* ~8-fold. Consistent with *CYP1A1* mRNA levels, 10 μM 5-HT induced ethoxyresorufin-O-deethylase (EROD) activity in a time-dependent manner with a ~3-fold induction after 8 h and a ~20-fold induction after 24 h. For subsequent experiments, 10 μM 5-HT was used since this concentration robustly induced CYP1A1 mRNA expression and activity at 8 h and 24 h, and is comparable to 5-HT concentration at the mucosal surface^33^.

To examine the mechanism by which 5-HT induces *CYP1A1* mRNA, we treated Caco-2 cells with 5-HT in the presence or absence of transcription inhibitor actinomycin D. *CYP1A1* mRNA induction was completely blocked in the presence of actinomycin D which suggests that 5-HT increases *CYP1A1* via a transcriptional mechanism (Fig. 1d).

### Role of serotonergic machinery in the induction of *CYP1A1* mRNA by 5-HT

#### 5-HT mediated CYP1A1 induction is 5-HTR independent

**Figure 2:**
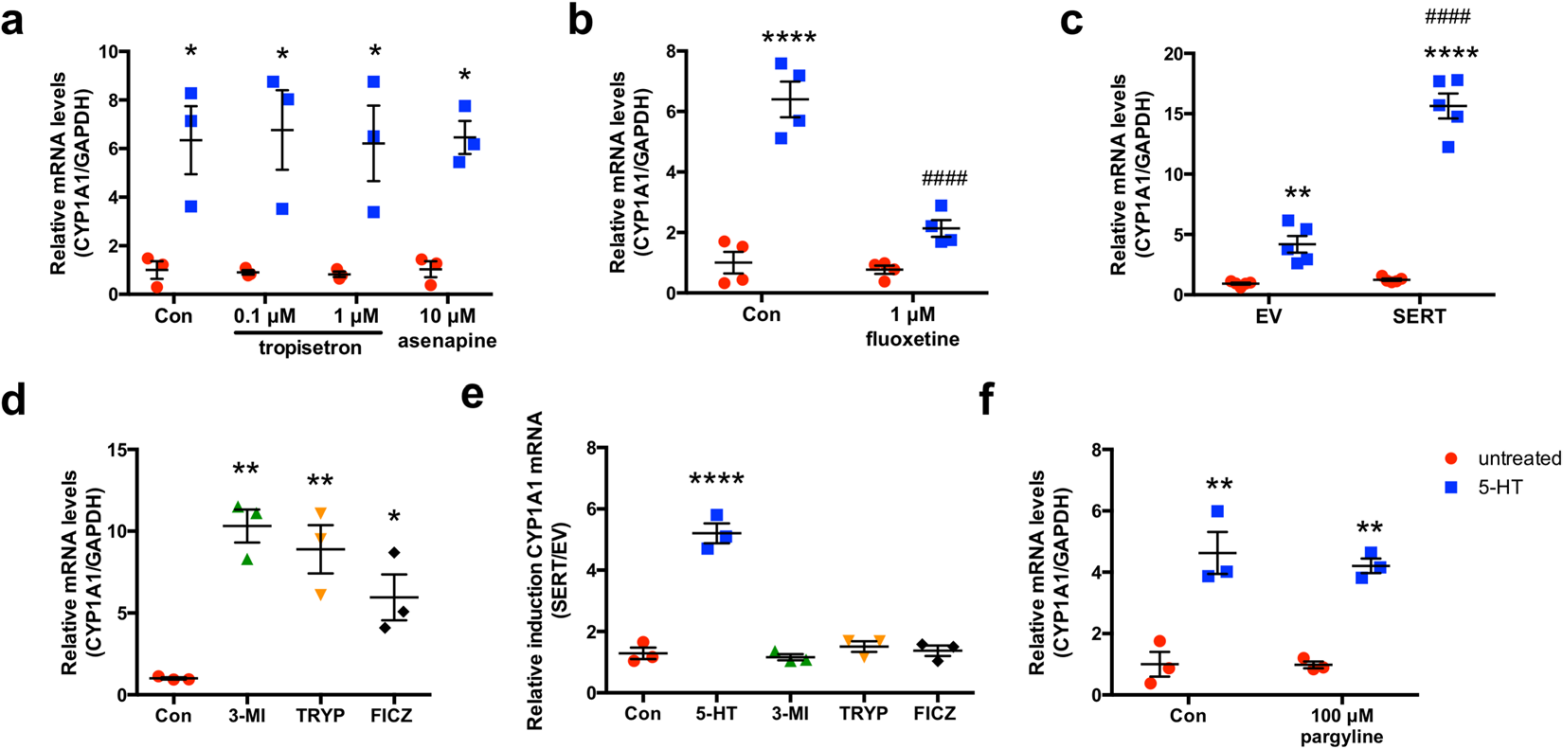
Induction of CYP1A1 mRNA is independent of 5-HTRs and MAOs but is dependent upon SERT. Caco-2 cells were plated at low density and allowed to differentiate for 10–14 d in medium containing 20% serum before treatments were performed without transfection (**a**,**b**,**f**). When transfection was performed, Caco-2 cells were treated beginning 24 h post-transfection (**c**–**e)**. (**a**) Caco-2 cells were treated with vehicle (Con) or 5-HTR antagonists tropisetron or asenapine apically for 1 h before co-treatment with 5-HT for 8 h in serum-free medium (*n* = 3). (**b**) Caco-2 cells were treated with vehicle (Con) or fluoxetine (1 μM) for 4 d in serum-containing medium before co-treatment of 5-HT (10 μM) with fluoxetine (1 μM) for 12 h in serum-free medium (*n* = 4). (**c**) Caco-2 cells were transiently transfected with SERT overexpression plasmid or empty vector (EV) by Amaxa electroporation before treatment with 5-HT (10 μM) for 24 h (*n* = 5). (**d**,**e**) Caco-2 cells were transfected with EV or SERT by Amaxa electroporation before treatment with vehicle (Con) or AhR ligands 3-methylindole (3-MI, 100 μM), tryptamine (TRYP, 100 μM), or 6-formylindolo[3,2*b*]carbazole (FICZ, 10 nM) for 24 h in serum-free medium. (**d**) depicts *CYP1A1* expression when transfected with EV while (**e**) depicts the ratio of *CYP1A1* induction between SERT and EV transfected cells, with no difference observed for any treatments other than 5-HT (*n* = 3). (**f**) Caco-2 cells were treated with vehicle (Con) or MAO inhibitor pargyline for 1 h before co-treatment with 5-HT for 8 h in serum-free medium (*n* = 3). Data represent the relative expression of *CYP1A1* mRNA quantified by qPCR. Data analyzed by 1-way (**a**–**c**,**f**) or 2-way (**d**,**e**) ANOVA followed by Tukey’s (**a**–**c**,**f**) or Bonferroni’s (**d**,**e**) multiple comparisons test. *P < 0.05, **P < 0.01, ****P < 0.0001 vs. untreated. ^###^*P* < 0.001 vs. no fluoxetine or EV. Red circle = untreated, blue square = 5-HT, green triangle = 3-MI, yellow triangle = TRYP treated, black diamond = FICZ.

Asenapine is a non-specific 5-HTR antagonist that is able to inhibit all classes of 5-HTRs expressed on intestinal epithelial cells except for 5-HT_3_ and 5-HT_4_, whereas tropisetron is a dual 5-HT_3_/5-HT_4_ antagonist at a concentration of 1 μM and a 5-HT_3_ specific antagonist at a concentration of 0.1 μM. Neither asenapine nor tropisetron were able to block the induction of *CYP1A1* mRNA by 5-HT. These findings suggest that 5-HT-mediated induction of *CYP1A1* is independent of 5-HTRs (Fig. 2a).

#### Induction of CYP1A1 mRNA expression by 5-HT is SERT-dependent

We next examined if *CYP1A1* mRNA induction by 5-HT was dependent upon SERT-mediated uptake of 5-HT. Caco-2 cells were treated with the selective serotonin reuptake inhibitor (SSRI) fluoxetine for 4 d prior to treatment with 5-HT^20,34^. *CYP1A1* mRNA induction by 5-HT was remarkably attenuated by fluoxetine pretreatment (Fig. 2b). In contrast, overexpression of SERT prior to treatment with 5-HT for 24 h was able to augment the induction of *CYP1A1* mRNA by 5-HT compared to empty vector (Fig. 2c). These data indicate that the effect of 5-HT on CYP1A1 is dependent upon uptake of 5-HT into the cell via SERT.

We next overexpressed SERT or empty vector and treated Caco-2 cells with known AhR ligands 3-methylindole (100 μM), tryptamine (100 μM), and the high potency ligand FICZ (10 nM). These ligands significantly induced *CYP1A1* mRNA in the cells transfected with empty vector (Fig. 2d). However, SERT overexpression did not affect the ability of these ligands to induce *CYP1A1* mRNA supporting the fact that the involvement of SERT on *CYP1A1* mRNA induction is specific to 5-HT (Fig. 2e).

#### Metabolism by MAO is not required for induction of CYP1A1 mRNA expression by 5-HT

To examine the role of MAO metabolism of 5-HT into 5-HIAA in the induction of CYP1A1 expression by 5-HT, Caco-2 cells were pretreated with pargyline, an irreversible MAO inhibitor before 5-HT treatment^20^. The presence of the inhibitor did not affect the ability of 5-HT to induce *CYP1A1* mRNA, suggesting that the effect is independent of metabolic conversion of 5-HT by MAO (Fig. 2f).

### Effect of 5-HT on CYP1A1 in a colonic crypt-derived cell line (T84)

**Figure 3:**
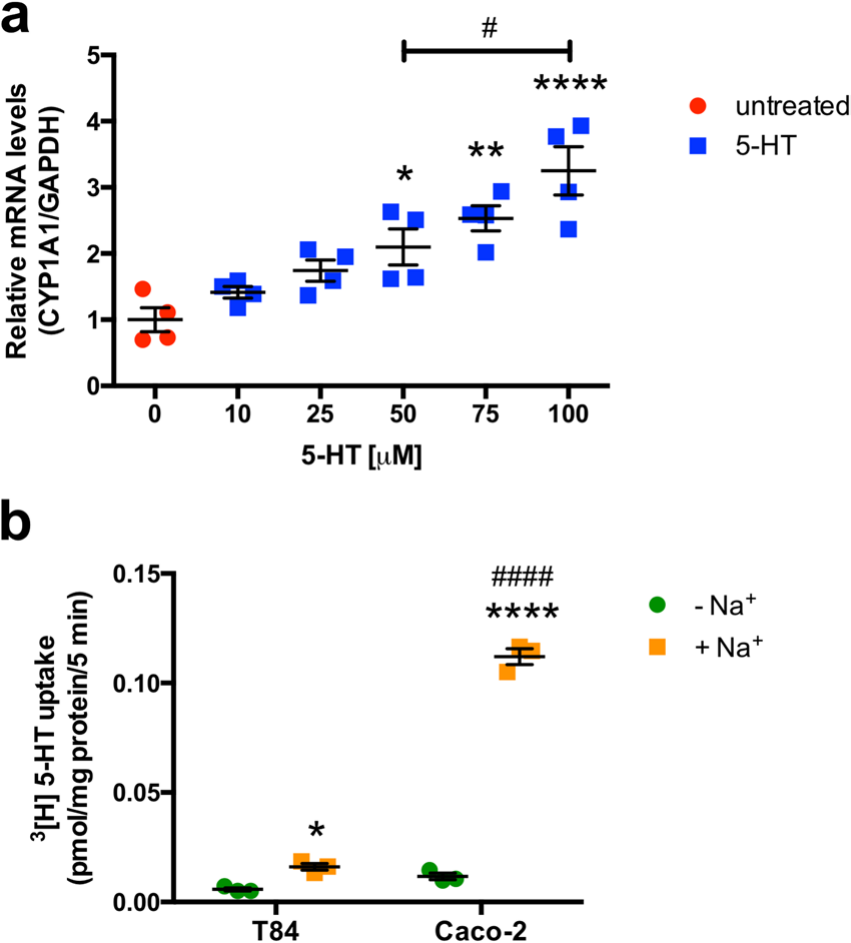
Effect of 5-HT on *CYP1A1* mRNA in T84 cells. (**a**) T84 cells were plated at low density and allowed to differentiate for 10–14 d in medium containing 5% serum before treatments were performed. T84 cells were treated for 24 h with 5-HT (10 μM–100 μM) in serum-free medium. Data represent the relative expression of *CYP1A1* mRNA quantified by qPCR (*n* = 4). (**b**) T84 and Caco-2 cells were plated at low density and allowed to differentiate for 10–14 d in medium containing 5% or 20% serum, respectively. SERT function was measured in T84 and Caco-2 cells as^3^ [H]5-HT uptake in the presence or absence of Na^+^ (*n* = 3). Data were analyzed by 1-way (**a**) or 2-way (**b**) ANOVA followed by Tukey’s multiple comparison’s test. *P < 0.05, **P < 0.01, ****P < 0.0001 vs. no 5-HT or −Na^+^. ^#^*P* < 0.05 and ^####^*P* < 0.0001 between groups. Red circle = untreated, blue square = 5-HT, green circle = −Na^+^, orange square = +Na^+^.

Caco-2 cells are derived from the large intestine, but functionally and morphologically represent enterocytes upon differentiation. On the other hand, the colonic crypt-derived cell line T84 has characteristics similar to colonocytes^35^. To determine if the effect of 5-HT on *CYP1A1* mRNA was cell-line specific, T84 cells were treated with varying concentrations of 5-HT (10–100 μM 5-HT). After 24 h, 5-HT was able to induce *CYP1A1* mRNA in T84 cells but to a much lesser extent than in Caco-2 cells (~3-fold with 100 μM 5-HT) (Fig. 3a).

To assess whether these differences in responsiveness to 5-HT correlates with differences in SERT function in Caco-2 vs T84 cell, the NaCl-sensitive [^3^H]5-HT uptake was measured. While both cell-lines exhibited [^3^H]5-HT uptake in a Na^+^ dependent manner, the uptake measured in T84 cells was remarkably less than in Caco-2 cells (Fig. 3b). These data suggest that the ability of 5-HT to induce *CYP1A1* mRNA in epithelial cells is positively correlated with their capacity to uptake 5-HT.

### Role of AhR and ARNT in the induction of CYP1A1 mRNA expression by 5-HT

#### Functional AhR and ARNT are required for induction of CYP1A1 mRNA expression by 5-HT

**Figure 4:**
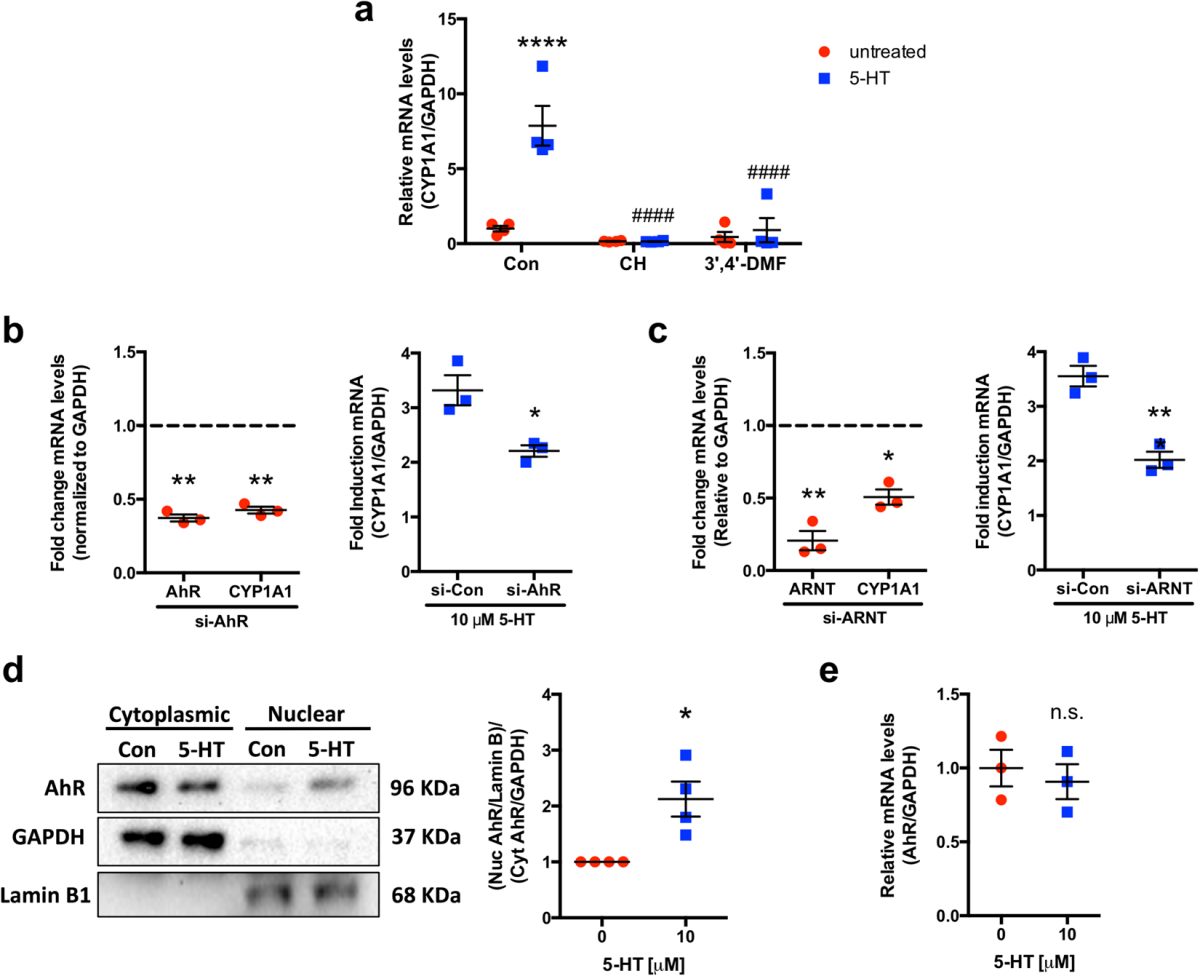
AhR activation is required for 5-HT to induce *CYP1A1* mRNA. Caco-2 cells were plated at low density and allowed to differentiate for 10–14 d in medium containing 20% serum before treatments were performed without transfection (**a**,**d**,**e**). When transfection was performed, Caco-2 cells were treated beginning 24 h post-transfection (**b**,**c**). (**a**) Caco-2 cells were treated with vehicle (Con) or AhR antagonists 10 μM CH-223191 (CH) and 10 μM 3′,4′-dimethoxyflavone (3′,4′-DMF) for 1 h before co-treatment of each antagonist with 5-HT (10 μM) for 24 h in serum-free medium. Data represent the relative expression of *CYP1A1* mRNA quantified by qPCR. Data were analyzed by 1-way ANOVA followed by Tukey’s multiple comparison’s test (*n* = 4). (**b**,**c**) Caco-2 cells were transfected with control siRNA (si-Con), *AhR* siRNA (si-AhR), or *ARNT* siRNA before treatment with 5-HT (10 μM) for 8 h (si-*AhR*) or 24 h (si-*ARNT*) in serum-free medium. *AhR*, *ARNT*, and *CYP1A1* mRNA expression were determined in untreated cells by qPCR and results are expressed as fold change compared to control siRNA. Data analyzed by paired 2-tailed Student’s *t*-test. Unpaired 2-tailed Student’s *t*-test was used to compare *CYP1A1* mRNA induction after 5-HT treatment in control siRNA vs. *AhR* or *ARNT* siRNA transfected cells (*n* = 3). (**d**) Caco-2 cells were treated with 5-HT (10 μM) for 4 h in serum-free medium before nuclear and cytoplasmic extracts were isolated and Western blotting was performed for AhR. Nuclear AhR was normalized to Lamin B1 and cytoplasmic AhR was normalized to GAPDH. The ratio between nuclear and cytoplasmic AhR as determined by densitometry was analyzed by paired 2-tailed Student’s *t*-test (*n* = 4). Full-length blots are shown in Supplementary Figure S1. (**e**) Caco-2 cells were treated with 5-HT (10 μM) for 24 h in serum-free medium. Data represent the relative expression of *AhR* mRNA quantified by qPCR (*n* = 3). *P < 0.05, **P < 0.01, ****P < 0.0001 vs. untreated, control siRNA, or no 5-HT. ^####^*P* < 0.0001 vs. 5-HT treatment without AhR antagonist. n.s. = not significant. Red circle = untreated, blue square = 5-HT.

To examine the dependence of *CYP1A1* mRNA induction by 5-HT on AhR, we treated Caco-2 cells with AhR antagonists CH-223191 (CH) and 3′,4′-dimethoxyflavone (3′,4′-DMF) prior to 5-HT treatment. In the case of both antagonists, *CYP1A1* mRNA induction was completely abolished (Fig. 4a). To complement the pharmacological inhibition, siRNA mediated knockdown of AhR and ARNT was utilized. A significant knockdown of both AhR (~60%) and ARNT (~80%) was achieved. The knockdown was functional since there was a significant decrease in basal *CYP1A1* mRNA expression (Fig. 4b,c). In addition, this knockdown significantly attenuated the ability of 5-HT to induce *CYP1A1* mRNA (Fig. 4b,c). These data indicate that both functional AhR and ARNT are required for the 5-HT mediated increase in *CYP1A1* mRNA.

#### Increased activation of AhR occurs after treatment with 5-HT

To establish whether AhR is translocating to the nucleus following treatment with 5-HT, we examined the changes in nuclear and cytoplasmic AhR following 5-HT treatment. The nuclear to cytoplasmic ratio of AhR was increased after treatment of Caco-2 cells with 5-HT for 4 h (Fig. 4d). To exclude the possibility that 5-HT is increasing AhR levels rather than activating AhR, we measured *AhR* mRNA after treatment with 5-HT and found that there was no difference in transcript levels after treatment (Fig. 4e).

### 5-HT induces XRE-mediated reporter activity

**Figure 5:**
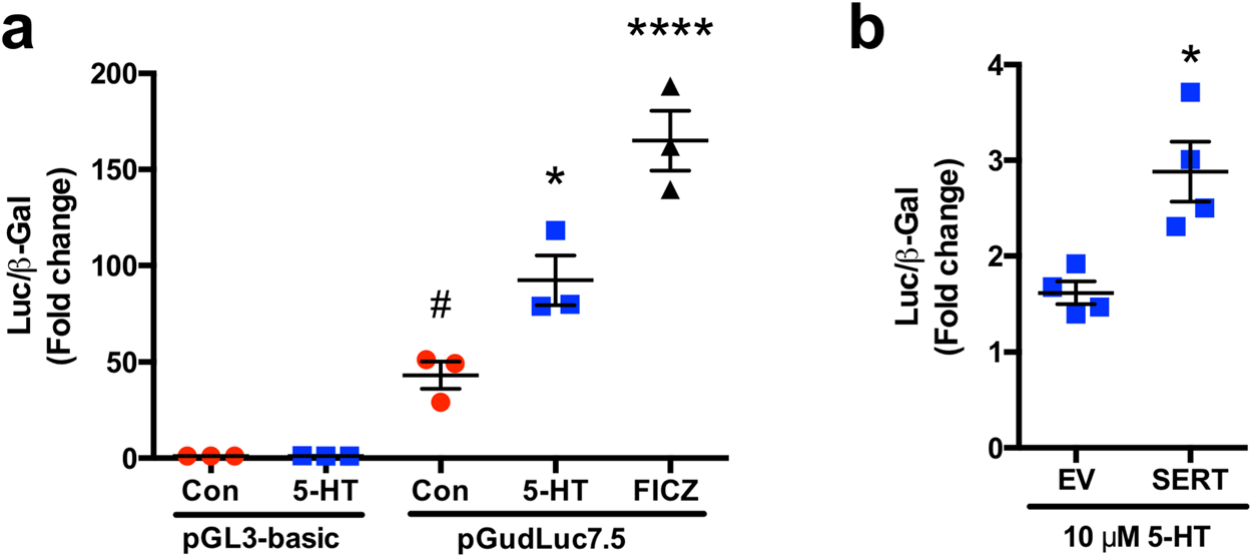
5-HT induces XRE reporter activity in a SERT-dependent manner. Caco-2 cells were treated beginning 24 h post-transfection. (**a**) Caco-2 cells were transiently co-transfected with pGudLuc7.5 luciferase XRE-reporter or pGL3-basic along with a *β*-gal mammalian expression plasmid (CMV-*β*) by lipofectamine. Cells were treated with 5-HT (10 μM), AhR ligand 6-formylindolo[3,2*b*]carbazole (FICZ) (10 nM), or vehicle (Con) for 24 h in serum-free medium. Reporter activity was measured by luciferase assay. Assays were performed using quadruplicate wells for each treatment and values were normalized to *β*-gal activity. Results are expressed as fold-change activity of untreated (Con) pGL3-basic transfected cells. Data analyzed by 1-way ANOVA followed by Tukey’s multiple comparisons test (*n* = 3). *P < 0.05, ****P < 0.0001 vs. untreated cells transfected with pGudLuc7.5 ^##^*P* < 0.05 vs. pGL3-basic. (**b**) Caco-2 cells were transiently co-transfected with pGudLuc7.5 Luciferase XRE-reporter, CMV- *β*, and either a SERT overexpression vector or empty vector (EV) by Amaxa electroporation. Cells were treated with 5-HT (10 μM) for 24 h in serum-free medium. Reporter activity was measured by luciferase assay. Assays were performed using quadruplicate wells for each treatment and values were normalized to *β*-gal. Results are expressed as fold-change over the activity of untreated cells. Data analyzed by unpaired 2-tailed Student’s *t*-test (*n* = 4). *P < 0.05 vs. EV. Red circle = untreated, blue square = 5-HT, black diamond = FICZ.

To examine the ability of 5-HT to induce XRE-mediated transcription, we utilized the pGudLuc7.5 luciferase reporter which is driven by a 480-bp fragment of the murine CYP1A1 promoter containing 4 XREs in their native orientation^36^. Treatment with 5-HT as well as FICZ significantly induced reporter activity relative to the reporter activity of untreated cells (Fig. 5a). Further, the induction of reporter activity by 5-HT was enhanced with SERT overexpression (Fig. 5b).

### Microarray analysis of SERT KO ileal mucosa

**Figure 6:**
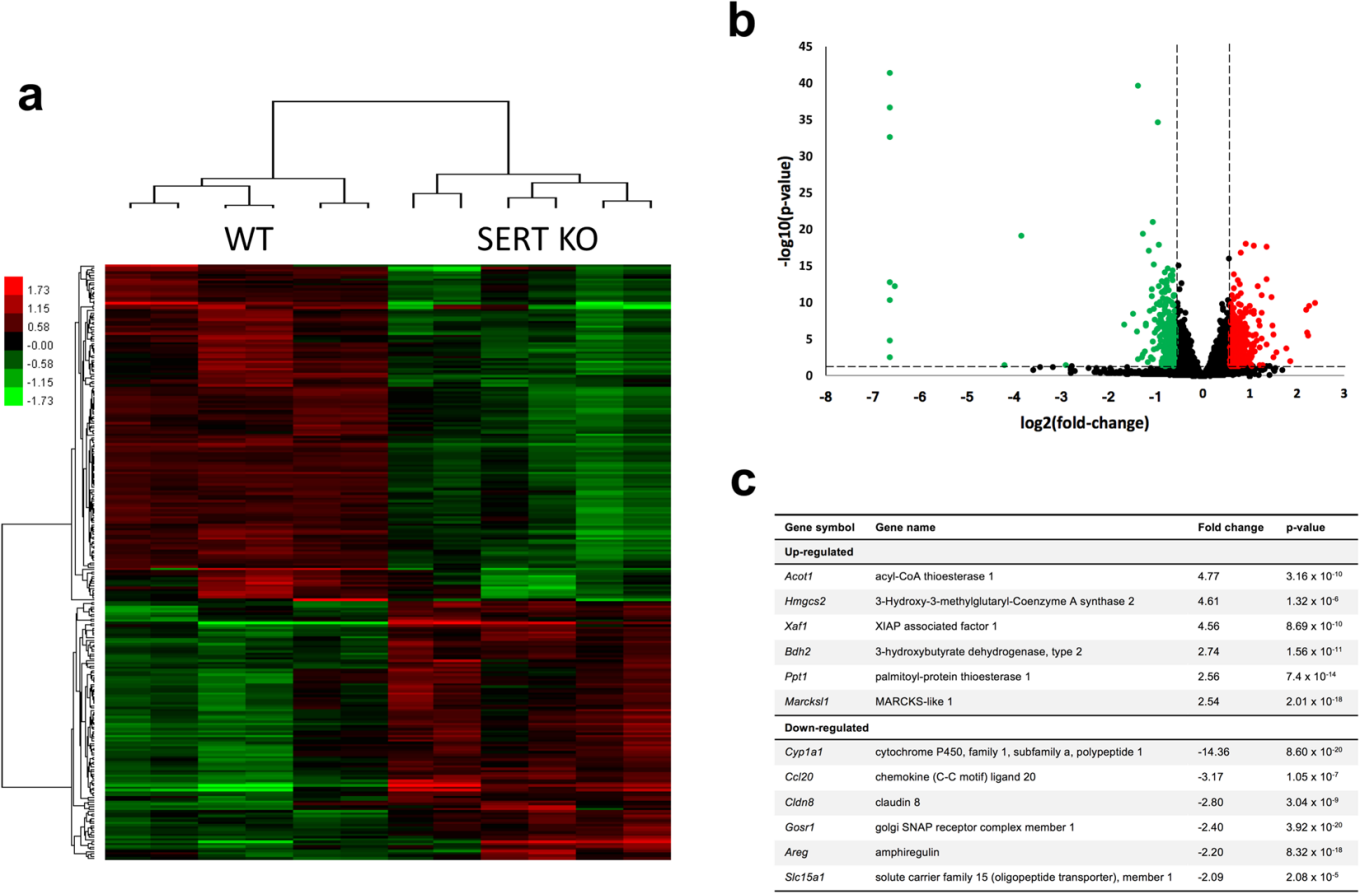
Microarray gene expression analysis of SERT KO ileal mucosa. Gene expression in the ileal mucosa of 6 WT and 6 SERT KO mice was analyzed by microarray analysis as described in Methods. (**a**) Heat map showing the result of unsupervised hierarchical clustering of selected differentially expressed genes between WT and SERT KO. (**b**) Volcano plot showing the distribution of differentially expressed genes with cutoff criteria of log2|fold-change| ≥ 0.585 and a p-value < 0.05. Up-regulated genes shown in red and down-regulated genes shown in green. (**c**) List of selected genes that were significantly up-regulated or down-regulated in SERT KO mouse ileal mucosa. Full list of differentially expressed genes available at GEO (accession GSE93534).

To validate our cell-culture based studies in a suitable *in vivo* model, we utilized WT mice and mice lacking a functional SERT (SERT KO). Microarray analysis was used to compare the gene expression profile of WT and SERT KO ileal mucosa in the absence of any overt inflammation. A total of 492 genes were up-regulated in SERT KO, and 292 genes were downregulated (Fig. 6). Of all of the genes measured, *Cyp1a1* had the greatest change with a 14.36-fold decrease in expression in SERT KO ileal mucosa. The second-most downregulated gene was *Ccl20* which encodes CCL20, an antimicrobial peptide and chemokine that has recently been found to contain several XREs in its promoter^28^. The AhR target gene *Areg*, which encodes the growth factor amphiregulin, was also down regulated in the microarray^29^. Of note, genes implicated in Crohn’s disease such as *Cldn8* (encodes tight junction protein claudin 8) and *Tnfsf15* were also downregulated in SERT KO mice^30–32,37^. Several genes involved in fatty acid metabolism and trafficking were up-regulated including *Acot1*, *Hmgcs2*, *Bdh2*, and *Ppt1*.

### CYP1A1 mRNA and protein expression is suppressed in SERT KO intestinal mucosa

**Figure 7:**
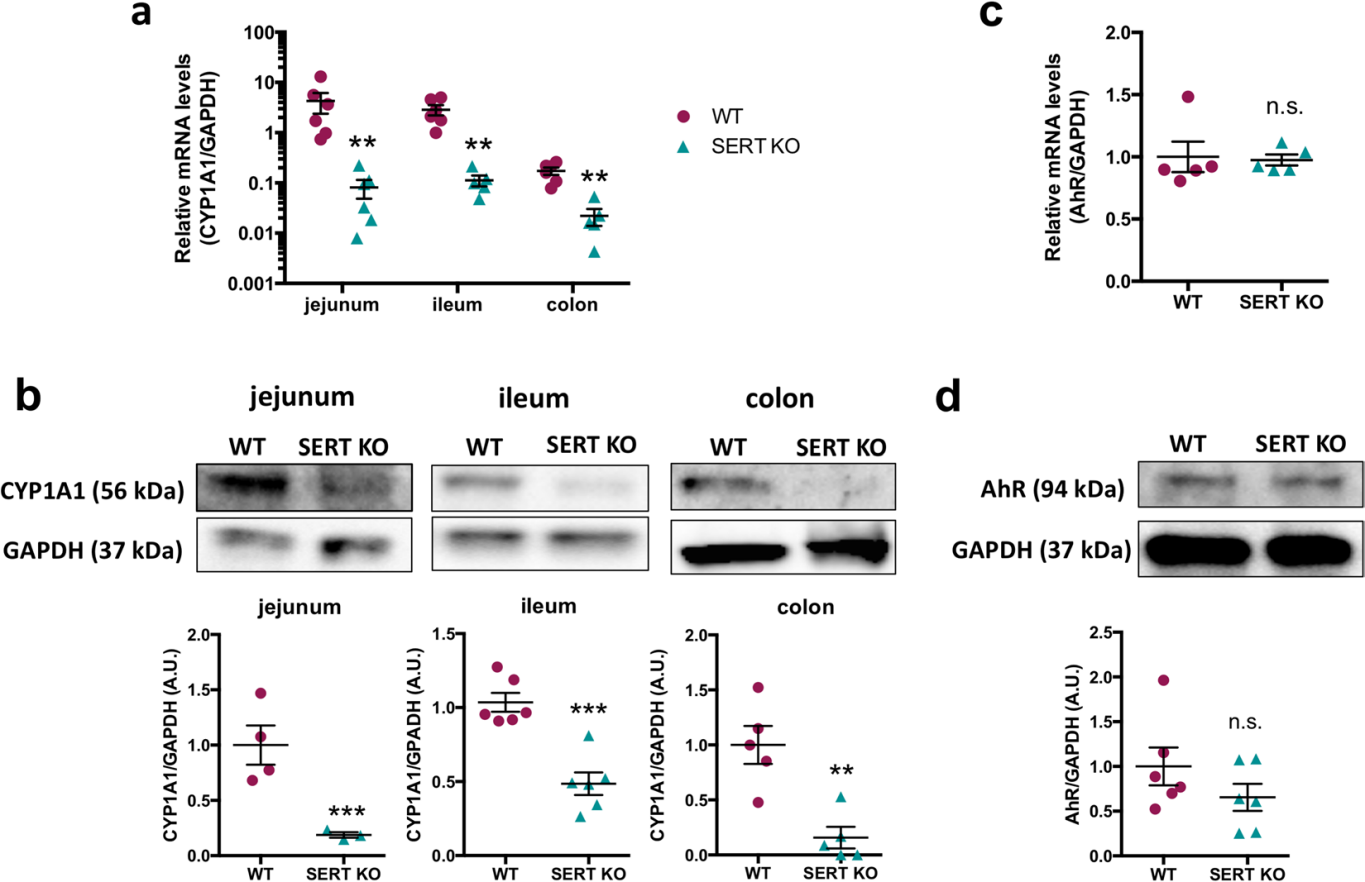
CYP1A1 expression is suppressed along the intestine of SERT KO mice. (**a**) Total RNA was isolated from intestinal mucosal scrapings from WT and SERT KO mice. Data represent the relative expression of *CYP1A1* mRNA quantified by qPCR. Data analyzed by Mann-Whitney *U* test (*n* = 5–6). **P < 0.01 vs. WT. (**b**) Protein lysates were prepared from intestinal mucosal scrapings from WT and SERT KO mice. CYP1A1 protein was measured by Western blot and normalized to GAPDH (*n* = 4–6). Full-length blots are shown in Supplementary Figure S2. (**c**) Total RNA was isolated from ileal mucosal scrapings of WT and SERT KO mice. Data represent the relative expression of *AhR* mRNA quantified by qPCR. (**d**) AhR protein was measured in the ileal mucosa by Western blot. Densitometric analysis of relative band intensities expressed as arbitrary units. Full-length blots are shown in Supplementary Figure S2. Data analyzed by 2-tailed unpaired Student’s *t*-test (*n* = 4–6). **P < 0.01, ***P < 0.001. n.s. = not significant. Magenta circle = WT, turquoise triangle = SERT KO.

To validate the microarray results, we measured mRNA expression of *Cyp1a1* along the length of the intestine of SERT KO mice by RT-PCR. Consistent with the microarray, basal *Cyp1a1* mRNA expression was suppressed in the jejunum, ileum, as well as the colon (Fig. 7a). CYP1A1 protein levels were also significantly decreased in SERT KO jejunum, ileum, and colon (Fig. 7b). AhR mRNA and protein levels in the ileal mucosa did not significantly differ between the WT and SERT KO mice (Fig. 7c,d) suggesting an impairment in the activation of AhR consistent with *in vitro* data.

## Discussion

The classic physiological functions of 5-HT in the GI tract mainly relate to initiation of peristalsis, visceral sensation, and controlling movement of fluid and electrolytes, which are mediated by activation of specific 5-HTR subtypes. Studies over the past decade have revealed a new role for 5-HT and SERT in the pathophysiology of intestinal inflammatory disorders, but the molecular mechanisms that contribute to this association are poorly understood^25–27,38^. Our present studies provide novel evidence that 5-HT activates the AhR pathway and induces CYP1A1 expression in IECs via a SERT dependent process. This was evident from key findings showing that (i) 5-HT concentration- and time-dependently increased *CYP1A1* mRNA levels and this increase was associated with increased CYP1A1 activity; (ii) *CYP1A1* mRNA induction by 5-HT was diminished by SERT inhibition via SSRI, fluoxetine; (iii) overexpression of SERT in Caco-2 cells augmented the induction of CYP1A1 expression by 5-HT; (iv) co-treatment of Caco-2 cells with AhR antagonists (CH-223191 and 3′,4′-DMF) as well as siRNA directed against AhR and ARNT attenuated the 5-HT induced increase in *CYP1A1* mRNA; (v) 5-HT treatment was associated with increased nuclear translocation of AhR with no change in AhR levels; (vi) 5-HT induced XRE reporter activity in Caco-2 cells in a SERT-dependent manner; (vii) mice with deletion of SERT exhibit drastic reduction in *Cyp1a1* expression in the mucosa of the small and large intestine; (viii) microarray analysis of SERT KO mice ileal mucosa revealed that multiple AhR responsive genes in addition to *Cyp1a1* were downregulated, including anti-microbial chemokine *Ccl20* and the growth factor *Areg*.

Our findings demonstrating a novel interaction of 5-HT with AhR, for the first-time report, that 5-HT activated an intracellular receptor in intestinal epithelial cells, as opposed to the membrane bound receptor family of 5HTRs, in which 5-HT binding occurs extracellularly. Several classes of 5-HTRs exist in the GI tract including 5-HT_1_, 5-HT_2_, 5-HT_3_, 5-HT_4_, and 5-HT_7_^39^. Caco-2 cells have been shown to express subclasses 5-HT_1A_, 5-HT_3_, 5-HT_4_, and 5-HT_7_^18,40^. 5-HT_1_, 5-HT_2_, 5-HT_4_, and 5-HT_7_ are coupled via guanine nucleotide binding (G) proteins to their effector molecules, whereas 5-HT_3_ is a ligand gated ion channel^18^. We found that co-treatment with asenapine (5-HT_1_, 5-HT_2_, 5-HT_5_, 5-HT_6_, 5-HT_7_ antagonist) or tropisetron (5-HT_3_, 5-HT_4_ antagonist) did not interfere with the ability of 5-HT to induce CYP1A1 expression. It is interesting to note that 5-HTR antagonists were recently shown to exacerbate intestinal inflammation rather than being protective, which undermines the role of extracellular 5-HT in the pathogenesis of intestinal inflammation^41^.

Intracellular 5-HT has previously been shown to have physiologic roles in non-intestinal tissues by serotonylation, which describes the covalent linkage of 5-HT to proteins as a post-translational modification^42,43^. This process is catalyzed by the enzyme transglutaminase, which covalently binds the amino group of 5-HT to the γ-carboxamide of glutamine^42,43^. Small G-proteins are the most common substrate for serotonylation, but other proteins including components of the cytoskeleton are also serotonylated^42,43^. Processes regulated by serotonylation include insulin release from pancreatic β-cells, activation of platelets during coagulation, and smooth muscle contraction^43–45^. Since our data indicate that the effect of 5-HT on CYP1A1 is dependent upon intracellular accumulation of 5-HT, we cannot exclude the involvement of serotonylation.

The ability of tryptophan metabolites to activate AhR has been previously studied^13,14,46,47^. For example, early reports show that both tryptamine and indoleacetic acid are AhR ligands, with a more recent study showing that activation of AhR by tryptamine requires metabolism by MAO^13,46^. Indole and its 3-substituted derivatives have also been shown to be AhR ligands,^14,15^ suggesting a role for bacterial metabolism of tryptophan in activating intestinal AhR. Interestingly, some indole derivatives that are established AhR ligands have been shown to be transported into the cell via SERT, particularly tryptamine which has an affinity for SERT similar to 5-HT^48^. However, we found that SERT overexpression did not affect the ability of these other derivatives to activate AhR. This might be due to the nonpolar nature of these molecules which allows entry into the cell in the absence of an active transport process.

While *CYP1A1* is considered a canonical AhR target gene, AhR-independent mechanisms have also been reported to regulate CYP1A1 expression^49^. However, the antagonism of AhR by both CH-223191 (CH), a ligand-selective AhR antagonist, and 3′,4′-dimethoxyflavone (3′,4′-DMF), a pure AhR antagonist that also exhibits antiestrogen properties, completely abolished the 5-HT induced increase in *CYP1A1*. With siRNA knockdown of AhR and ARNT, there was a partial inhibition of the induction of *CYP1A1*. Although functional knockdown of AhR and ARNT was achieved, there was likely still enough expression of each protein to allow 5-HT to induce *CYP1A1*, although the effect was attenuated. This has also been observed in other studies utilizing siRNA to study AhR signaling, in which established AhR ligands were able to induce *CYP1A1* mRNA in the situation of incomplete knockdown of AhR or ARNT^14,50^.

Only two previous studies have examined 5-HT in the context of AhR activation. The first study utilized an XRE-luciferase reporter in yeast and found that 5-HT does not induce reporter activity, which is not surprising because yeast lack a transporter for 5-HT^47^. The second study performed electrophoretic mobility shift to observe XRE-binding after incubation of hepatic cytosol with 5-HT^13^. A shift was not observed, suggesting that 5-HT does not bind AhR directly. While MAO inhibition did not affect the ability of 5-HT to induce CYP1A1 expression, it is possible that 5-HT metabolites from other pathways, such as the melatonin pathway, may interact with AhR. Another possibility is that an intestine-specific factor is required for an interaction between AhR and 5-HT. In this regard, our data utilizing an XRE-responsive reporter in intestinal epithelial cells showed that 5-HT is able to increase AhR activity, which was enhanced with increased SERT expression. We found that 5-HT was able to induce *CYP1A1* mRNA in intestinal epithelial cell lines, where the magnitude of induction correlated with SERT function. Therefore, 5-HT is an activator of AhR in SERT-expressing intestinal epithelial cells.

It is possible that 5-HT could be a direct ligand of AhR or may act via indirect mechanisms, which will be the subject of future investigations. Several reports have suggested that interference with the metabolic clearance of endogenous ligands, such as FICZ, is an alternative mechanism for activation of AhR by a molecule rather than direct binding of the molecule itself to AhR^51^. Further intensive studies are needed to provide insight into whether 5-HT is a direct or indirect activator of AhR.

In mice lacking SERT, it is not clear which cell types are responsible for the increased susceptibility of these mice to inflammation. It is speculated that loss of SERT will increase the extracellular availability of 5-HT, which will be able to bind to immune cells in the lamina propria to promote inflammation. This concept is undermined by the fact that total colonic 5-HT content does not increase in SERT KO mice^27^. Further, studies that examine the effects of 5-HT on immune cells *in vitro* are inconsistent. For example, it has been reported that 5-HT activates dendritic cells via binding to 5-HT_7_ receptors to increase cytokine production^52^. In contrast, other studies highlight that 5-HT_7_ receptors on dendritic cells are actually anti-inflammatory^53^. While the current studies were performed in intestinal epithelial cells, many immune cells also express the machinery required to synthesize and uptake 5-HT^54^. It is possible, therefore, that 5-HT could be influencing AhR activation in intestinal immune cells as well. Activation of AhR in immune cells is known to facilitate IL-22 production from these cells, which is considered an important mechanism by which AhR activation counteracts inflammation^55^. IL-22 binds to receptors on epithelial cells to promote epithelial regeneration and proliferation, maintain the mucosal barrier, and increase production of antimicrobial peptides^56^. Extensive future studies examining the contribution of epithelial cells and immune cells to the enhanced susceptibility to inflammation observed in SERT KO mice should address this issue.

Our microarray gene expression analysis revealed the differential regulation of numerous genes in SERT KO ileal mucosa under basal conditions in the absence of any inflammation. Interestingly, the well-established IBS and Crohn’s Disease susceptibility gene *Tnfsf15*, was differentially expressed in SERT KO^30,31^. A recent GWAS study identified both *AhR* and *CCL20* as IBD susceptibility genes, underscoring the significance of our findings^31^. The decreased expression of *Cldn8* in SERT KO mice could also contribute to the pathogenesis of the enhanced inflammation observed in SERT KO mice. Increased expression of enzymes involved in fatty acid metabolism and trafficking in SERT KO ileal mucosa may be related to the metabolic disorder that emerges in these mice as they age^22^. More studies are needed to investigate the role of SERT deficiency in the differential expression of these genes.

The therapeutic potential of AhR ligands for the treatment of intestinal inflammatory disorders has been gaining attention over the past decade^9,10,57^. However, persistent activation of AhR in extra-intestinal tissues may lead to undesirable outcomes. The toxicity of 2,3,7,8-tetrachlorodibenzo-p-dioxin (TCDD), which causes an array of health problems including cancer, chloracne, and developmental abnormalities, can be entirely contributed to activation of AhR^58^. Thus, increased understanding of endogenous and tissue-specific mechanisms that govern AhR activity is essential to exploit AhR as a therapeutic target. Our studies indicate that increasing intestinal SERT expression may allow selective activation of the AhR pathway via increasing intracellular 5-HT to treat gut disorders where SERT expression is decreased.

**Figure 8:**
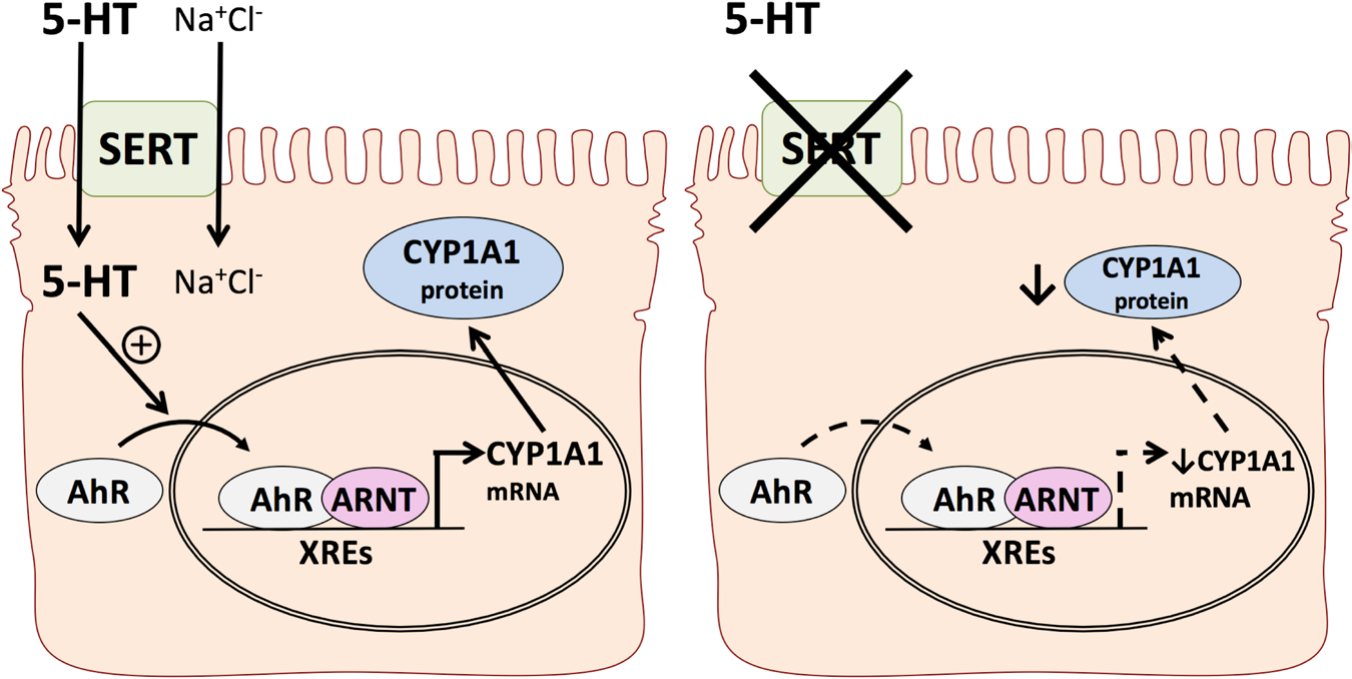
Proposed model of interactions between the serotonergic and AhR signaling pathways. In the intestinal mucosa of WT mice (left), enterocytes express SERT which is able to transport 5-HT into the cell via a Na^+^Cl^−^ dependent process. Once inside the cell, 5-HT is able to activate AhR to induce nuclear translocation, dimerization with ARNT, and binding to XREs on AhR target genes. This will induce transcription of target genes such as *Cyp1a1* which will be translated to protein. When SERT is absent as in SERT KO mice (right), enterocytes are not able to efficiently transport 5-HT into the cell. This will result in reduced AhR activation, and subsequently reduced expression of AhR target genes including *Cyp1a1*.

In conclusion, our findings provide a novel mechanism by which AhR is activated by an endogenous molecule, 5-HT, in intestinal epithelial cells. As depicted in Fig. 8, we conclude that in the presence of SERT, 5-HT is able to enter enterocytes via a Na^+^Cl^−^ dependent process. This intracellular 5-HT induces activation of AhR, which will then translocate to the nucleus and dimerize with ARNT. This dimer can bind to XRE regions on the promoters of AhR-responsive genes such as *CYP1A1* to induce their expression. In cases where SERT is absent or down-regulated such as in intestinal inflammation, less 5-HT will be able to enter the cell and the expression of AhR-responsive genes will be decreased (Fig. 8).

This mechanism linking the serotonergic system to AhR signaling contributes significantly to our understanding of how AhR, a critical mediator between the environment, the microbiota, and inflammation, is regulated under physiological and pathophysiological conditions. Maintaining appropriate levels of AhR activation is crucial for the survival and proliferation of both immune cells and epithelial cells in the intestinal mucosa in the absence of disease. In cases of inflammation, AhR is essential for the production of anti-inflammatory cytokines and their receptors to facilitate a robust response to pathogens, as well as proper mucosal healing and recovery. Enhancing our knowledge of how AhR is regulated will allow for the targeting of AhR as a therapy for patients suffering from enteric infections or IBD.

## Methods

### Chemicals

Serotonin hydrochloride, fluoxetine hydrochloride, pargyline hydrochloride, actinomycin D, CH-223191, 3-methylindole, tryptamine hydrochloride, and 3′,4′-dimethoxyflavone were obtained from Sigma. Asenapine maleate, tropisetron hydrochloride, and 6-formylindolo[3,2*b*]carbazole were obtained from Tocris. 7-ethoxyresorufin was obtained from Santa-Cruz.

### Animals

6 C57BL/6 J WT mice and 6 SERT KO mice of C57BL/6 J background (male mice aged 8–10 wk, 20–25 g) were purchased from Jackson Laboratory. The mice were housed in autoclaved polypropylene cages with corncob bedding. The mice had free access to food (Teklad Irradiated LM-485 Mouse/Rat Diet 7912, Envigo) and water and a 12 h light/dark cycle. After euthanasia, intestines were immediately resected and separated into jejunum, ileum, and colon. Mucosa was scraped and flash frozen in liquid nitrogen and stored at −80 °C for RNA extraction and lysate preparation.

### Microarray analysis

RNA extracted from the ileal mucosa of 6 WT mice and 6 SERT KO mice were pooled into pairs to create 3 samples per genotype. Each pooled sample containing 1 μg total RNA was first converted into cDNA. *In vitro* transcription was used to convert cDNA into amplified antisense RNA (aRNA), which was labeled with Cyanine 5-CTP (Cy5) fluorescent dye to generate Cy5-coupled aRNA target for Phalanx Biotech whole genome microarray system. The aRNA was fragmented into 50–200 bases for use on the microarray. Phalanx OneArray^TM^ Hybridization was performed overnight at 50 °C. The hybridized microarrays were scanned using an Agilent Microarray Scanner (G2505C) and raw data was extracted from the images using GenePix^TM^ 4. Data was analyzed using the Rosetta ResolverR System.

### Cell culture and treatments

Caco-2 cells and T84 cells were obtained from American Type Culture Collection (ATCC). Caco-2 cells were grown routinely in Eagle’s minimum essential medium (EMEM) (ATCC) supplemented with 20% fetal bovine serum (FBS) while T84 cells were grown routinely in Dulbecco’s modified Eagle’s medium: nutrient mixture F-12 (DMEM:F12) (ATCC) supplemented with 5% FBS. Caco-2 and T84 cells were maintained in 5% CO_2_ −95% air at 37 °C. Caco-2 and T84 cells were plated on 24-well plates (Corning) on plastic supports at a density of 2 × 10^4^ cells/well. Caco-2 and T84 cells were maintained 10–14 d post-plating to allow differentiation as previously described before treatments were performed for all experiments unless indicated^59^. Fluoxetine (selective serotonin reuptake inhibitor, 1 μM) was given for 4 d in EMEM supplemented with 20% FBS prior to co-treatment of 5-HT (10 μM) and fluoxetine (1 μM), which was given in serum-free EMEM. All other treatments were given in serum-free EMEM for Caco-2 and serum-free DMEM:F12 for T84. Cells were treated with asenapine or tropisetron (5-HT receptor antagonists), pargyline (MAO inhibitor), actinomycin D (transcription inhibitor), or AhR antagonists CH-223191 and 3′,4′-dimethoxyflavone for 1 h prior to the addition of 5-HT (10 μM). Cell lines were routinely tested for mycoplasma using a commercially available kit (Lonza) according the manufacturer’s instructions.

### [^3^H]Serotonin Uptake

Uptake of [^3^H]5-HT by Caco-2 cells and T-84 cells under unstimulated conditions was measured by assessing Na^+^- and Cl^−^ dependent [^3^H]5-HT uptake as previously described by us^60^. [^3^H]5-HT uptake was initiated by the addition of 300 μl of uptake buffer containing 25–50 nM [^3^H]5-HT (Perkin Elmer) for a time period of 5 min (linear range of uptake). The uptake was stopped by washing twice with ice-chilled 1X PBS. The cells were lysed completed by 500 μl 0.5 N NaOH overnight at 4 °C. Radioactivity was measured with a liquid scintillation counter (Packard) and relative protein levels were measured by Bradford (Bio-Rad).

### Transient transfection and luciferase assays

For SERT overexpression experiments, Caco-2 cells were transiently transfected by electroporation utilizing Amaxa Nucleofector T (Lonza) as described^61^. For transient transfection experiments without SERT overexpression, Caco-2 cells were transiently co-transfected with pGudLuc7.5 XRE luciferase reporter plasmid (kind gift from Dr. Michael Denison, University of California, Davis) and CMV-*β*, a *β*-gal mammalian expression plasmid (BD Biosciences) using lipofectamine 2000 transfection reagent (Invitrogen) as recommended by the manufacturer. Reporter experiments in which CMV-SERT was overexpressed, pGudLuc7.5 and CMV-*β* were co-transfected with either EV or CMV-SERT by electroporation utilizing Amaxa Nucleofector T (Lonza). Post-transfection (24 h), cells were treated for 24 h with 5-HT or AhR agonists. Activities of luciferase and *β*-gal were measured by a luminometer (Promega), utilizing kits from Promega and Clontech, respectively, according the manufacturer’s instructions. Reporter activity was calculated as a ratio of the luciferase activity to the *β*-gal activity for each sample. Each experiment was performed in quadruplicate wells, and repeated at least 3 times.

### siRNA-mediated silencing

For siRNA studies, Caco-2 cells were plated on 12-well plates at a density of 4 × 10^4^ cells/well, 24 h before transfection. After 24 h, Caco-2 cells were transfected with 40 pmol of *AhR* (sense: 5′-GGAUUAAAUUAGUUUGUGATT-3′, antisense: 5′-UCACAAACUAAUUUAAUCCAA-3′) or *ARNT* (sense: 5′-GGAACAAGAUGACAGCCUATT-3′, antisense: 5′-UAGGCUGUCAUCUUGUUCCGT-3′) specific siRNA and Allstars negative control siRNA (Qiagen) utilizing lipofectamine 2000 transfection reagent (Invitrogen) as recommended by the manufacturer. For *AhR* siRNA, cells were treated for 8 h with 5-HT (10 μM) beginning 24 h post-siRNA transfection. For *ARNT* siRNA, cells were treated 24 h with 5-HT (10 μM) beginning 24 h post-siRNA transfection. Silencing was validated by real time RT-PCR utilizing *AhR* or *ARNT* specific primers and target gene *CYP1A1* specific primers.

### RNA extraction and real time RT-PCR

RNA was extracted from mouse tissue using TRIzol (Fisher) followed by RNEasy column purification (Qiagen). RNA was extracted from cells using RNEasy column purification. Quantitative RT-PCR was performed using SYBR Green fluorescence as previously described^62^. The gene-specific primer sequences are listed in Supplementary Table S1. The relative mRNA levels were normalized to *GAPDH* mRNA levels using the ΔΔCt method.

### Ethoxyresorufin-O-deethylase Assay

The CYP1A1-dependent ethoxyresorufin-O-dethylase (EROD) activity of Caco-2 cells was assayed by first treating the cells with 5-HT in serum-free media for the indicated time point. The treatment media was removed and washed with 1X PBS before 300 μl of 50 mM NaHPO_4_ pH 8.0 containing 2 μM 7-ethoxyresorufin was added to each well of a 24-well plate. The cells were incubated at 37 °C for 20 min before termination of the reaction by removal of the medium. Medium was transferred to a 96-well plate and formation of resorufin was quantified on a multiwell plate reader in triplicate with the excitation/emission wavelengths of 544/590. The activity was expressed relative to the amount of protein present as determined by Bradford Assay (BioRad) according to the manufacturer’s protocol.

### Western blotting

Tissue lysates were prepared from mucosal scrapings using RIPA lysis buffer (Cell Signaling) supplemented with fresh protease inhibitor cocktail (Roche). Tissue was lysed by sonication, and the lysate was centrifuged at 13,000 g for 10 min at 4 °C. Nuclear and cytoplasmic extracts from Caco-2 cells were prepared using the NE-PER kit (Thermo) according to the manufacturer’s instructions. Protein concentration was measured by the method of Bradford (Bio-Rad). Equal amounts of tissue lysates were run on a 7.5% SDS-PAGE gel and then transferred onto a nitrocellulose membrane. Blocking was performed in 5% nonfat dry milk in 1X PBS for 1 h. The membranes were probed overnight at 4 °C. Primary antibodies used were anti-CYP1A1 (Santa Cruz Biotechnology: sc-20772) diluted 1:200, anti-AhR (Santa Cruz Biotechnology: sc-5579) diluted 1:200, anti-GAPDH (Sigma: G8795) diluted 1:3,000, and anti-Lamin B1 (abcam: ab16048) diluted 1:500. The membranes were washed three times with 1X PBS 0.1% Tween 20 for 10 min. The membranes were then probed with horseradish peroxidase conjugated goat anti-rabbit IgG secondary antibody (Santa Cruz) diluted 1:2,000 in 1% nonfat dry milk 1X PBS for 1 h. The membranes were washed three times with 1X PBS 0.1% Tween 20 for 10 min again before bands were visualized with enhanced chemiluminescence (ECL) detection reagents (Bio-Rad). Images were acquired by Imagelab 5.0 (Bio-Rad) and band intensities were measured using ImageJ software.

### Statistics

Microarray data was loaded into Rosetta Resolver® System (Rosetta Biosoftware) to process data analysis. The error in the measurements due to random factors and systemic biases was estimated by the Rosetta error model^63^. Repeated probes within one chip were averaged and median scaling was performed on the data to normalize the intensities. Two technical replicates were averaged before pair-wise comparison and error-weighted modeling were performed comparing data from WT and SERT KO. Selection criteria for differentially expressed genes were 2-fold change, P < 0.05, and an intensity difference between the two groups of ≥1000. Unsupervised hierarchical clustering analysis was performed by an average-link clustering algorithm carried out on selected differentially expressed gene lists after data transformation and mean centering. For all other experiments, 1-way or 2-way ANOVA followed by Tukey’s or Bonferroni’s multiple comparisons test, Mann-Whitney *U* test, or 2-tailed unpaired or paired Student’s *t*-test were utilized for statistical analysis as indicated with alpha set to 0.05. Values are means ± SEM and all experiments were performed in at least triplicate on ≥3 separate occasions. *p < 0.05, **p < 0.01, ***p < 0.001, ****p < 0.0001, ^#^*p* < 0.05, ^##^*p* < 0.01, ^###^*p* < 0.001, ^####^*p* < 0.0001 for comparisons as indicated. Analyses were performed using GraphPad Prism (Prism 6).

### Study approval

Animal studies were approved by the Animal Care Committees of the University of Illinois at Chicago and the Jesse Brown Veterans Affairs Medical Center. All studies were conducted in accordance with institutional guidelines and regulations at the University of Illinois at Chicago and the Jesse Brown Veterans Affairs Medical Center.

### Data availability

The microarray dataset generating during the current study are available in the Gene Expression Omnibus (GEO) repository (Accession GSE93534): https://www.ncbi.nlm.nih.gov/geo/query/acc.cgi?acc=GSE93534.

## Electronic supplementary material

Supplementary Information
