# Supplementary material for: Serotonin is an endogenous regulator of intestinal CYP1A1 via AhR
Source: Sci Rep. 2018 Apr 17;8:6103. doi: 10.1038/s41598-018-24213-5 (PMC5904159; doi:10.1038/s41598-018-24213-5)
Supplement: Supplementary file 1 — Supplementary Information [file 41598_2018_24213_MOESM1_ESM.pdf]

Supplementary Information

Serotonin is an endogenous regulator of intestinal CYP1A1 via AhR

Christopher Manzella[3], Megha Singhal[1], Waddah A Alrefai[1,2], Seema Saksena[1,2], Pradeep K Dudeja[1,2] and Ravinder K Gill[1,2*]

[1]Division of Gastroenterology & Hepatology, University of Illinois at Chicago, Chicago, IL, United States

[2]Jesse Brown VA Medical Center, Chicago, IL, United States

[3]Department of Physiology & Biophysics, University of Illinois at Chicago, Chicago, IL, United States

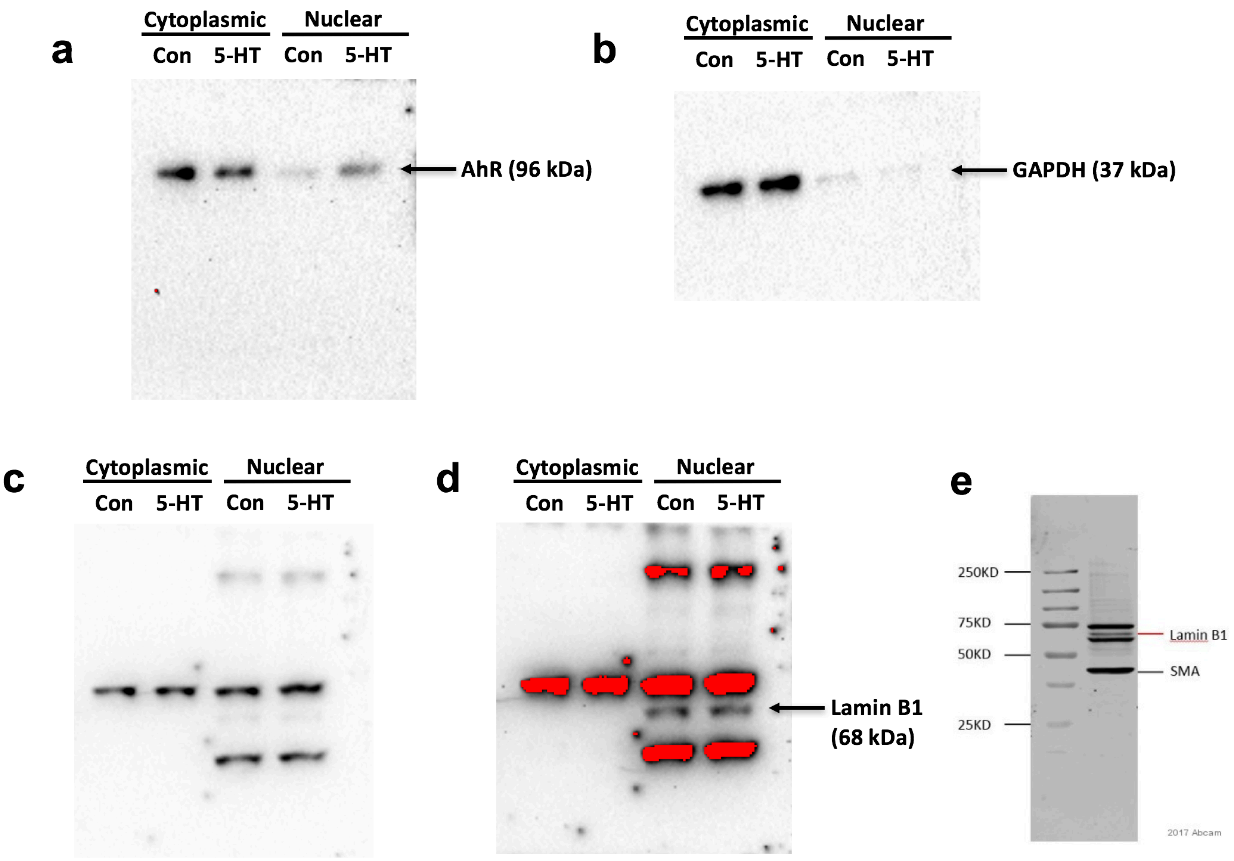

Supplementary Figure S1: Full-length Western blots for nuclear localization of AhR after 5-HT treatment

Caco-2 cells were treated with 5-HT (10 μM) for 4 h before nuclear and cytoplasmic extracts were isolated and Western blotting was performed. Full-length blots for the representative experiment displayed in the main text (Fig. 4d) are shown. Overexposure is shown in red. **(a)** Western blot for AhR. **(b)** Western blot for GAPDH. **(c)** Low-exposure blot for Lamin B1. **(d)** High-exposure blot for Lamin B1. **(e)** Image taken from Abcam identifying Lamin B1 band (http://www.abcam.com/lamin-b1-antibody-nuclear-envelope-marker-ab16048.html).

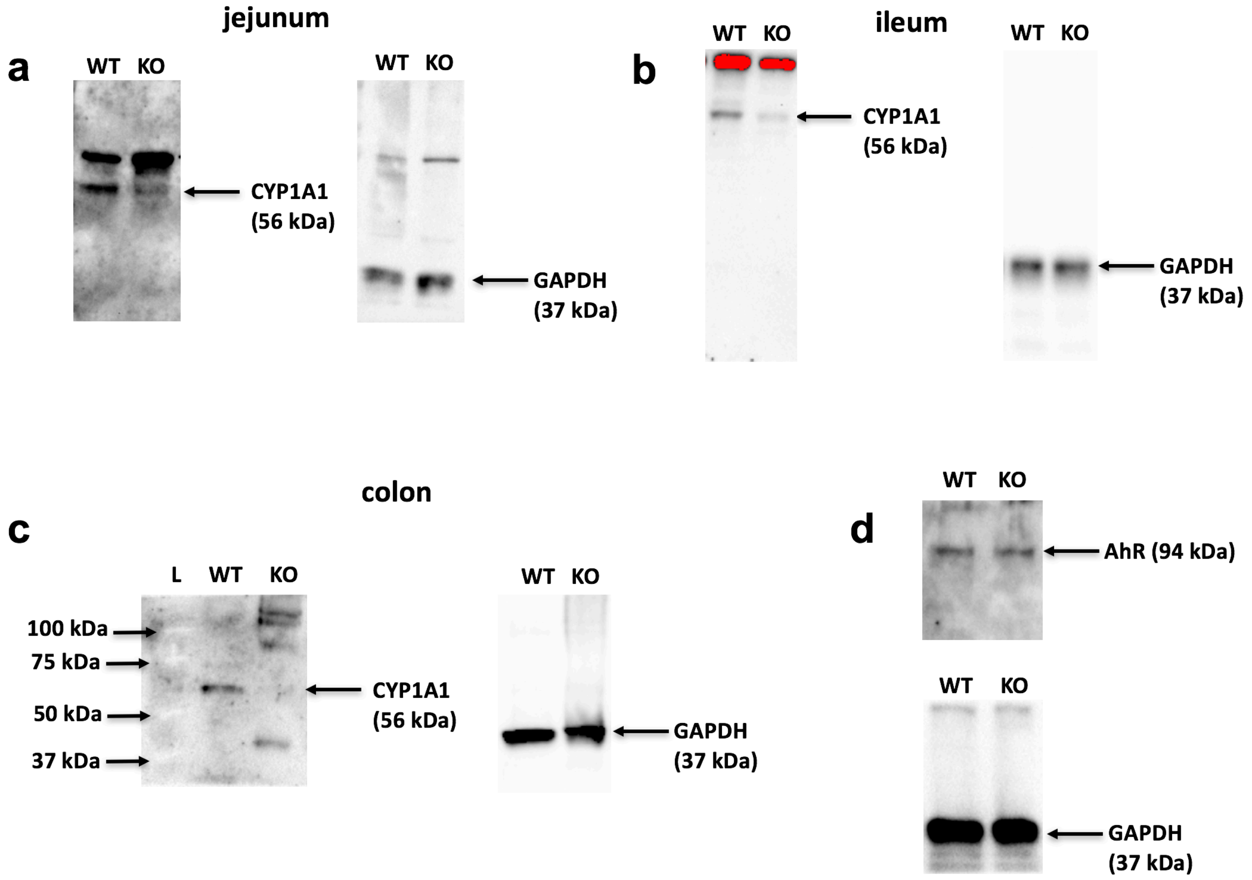

Supplementary Figure S2: Full-length Western blots for CYP1A1 and AhR expression in WT and SERT KO intestine

Protein lysates were prepared from intestinal mucosal scrapings from WT and SERT KO mice and Western blotting was performed. Full-length blots for the representative blots displayed in the main text (Fig. 7b,d) are shown. Overexposure is shown in red. **(a)** Western blots for CYP1A1 and GAPDH in jejunum. **(b)** Western blots for CYP1A1 and GAPDH in ileum. **(c)** Western blots for CYP1A1 and GAPDH in colon. Ladder (L) is shown with size markers labeled. **(d)** Western blots for AhR and GAPDH in ileum.

Supplementary Table S1. Gene-specific primers used for Real-Time PCR

Gene	Species	Accession No.	Primer sequence (5'→3')
CYP1A1	Human	NM_000499	F: TCGGCCACGGAGTTTCTTC<br>R: GGTCAGCATGTGCCCAATCA
GAPDH	Human	NM_001101.3	F: GAAATCCCATCACCATCTTCC<br>R: AAATGAGCCCCAGCCTTCT
AHR	Human	NM_001621	F: CAAATCCTTCCAAGCGGCATA<br>R: CGCTGACCTAAGAACTGAAAG
ARNT	Human	NM_001668	F: AACCTCACTTCGTGGTGGTC<br>R: CAATGTTGTGTCGGGAGATG
Cyp1a1	Mouse	NM_001136059	F: GGGTTTGACACAGTCACAACT<br>R: GGGACGAAGGATGAATGCCG
Gapdh	Mouse	NM_008084	F: TGTGTCCGTCGTGGATCTGA<br>R: CCTGCTTCACCACCTTCTTGAT
Ahr	Mouse	NM_013464	F: AGCCGGTGCAGAAAACAGTAA<br>R: AGGCGGTCTAACTCTGTGTTC
